# Toward clinical integration of generative AI in mental health: personalization, multimodality and inter-entity experience

**DOI:** 10.3389/fpubh.2026.1603238

**Published:** 2026-03-24

**Authors:** Fabio Frisone, Chiara Pupillo, Chiara Rossi, Giuseppe Riva

**Affiliations:** 1 Humane Technology Laboratory, Catholic University of the Sacred Heart, Milan, Italy; 2 Sophia University Institute, Figline e Incisa Valdarno (Firenze), Italy; 3 Department of Computer Science, University of Pisa, Pisa, Italy; 4 Department of Human Sciences, Guglielmo Marconi University, Rome, Italy; 5 Applied Technology for Neuro-Psychology Laboratory, IRCCS Istituto Auxologico Italiano, Milan, Italy

**Keywords:** clinical psychology, general psychology, generative artificial intelligence, mental health, psychological support, virtual reality

## Abstract

The integration of conversational agents (CAs) into mental health care presents a promising yet complex frontier. These systems have demonstrated potential in expanding access, enhancing user engagement and providing scalable support, particularly in contexts where human clinicians are unavailable. Despite these advantages, CAs face two fundamental limitations: their quality of interaction and the ability to facilitate self-reflection. This perspective article provides a theoretical framework with clinical relevance, identifying the critical challenges that must be addressed to transition Generative Artificial Intelligence (GenAI) from a conversational tool to an adjunctive psychological support. Firstly, personalization is essential. Advances in retrieval-augmented generation (RAG), fine-tuning, and emotional reasoning are necessary to enable context-aware and ethically grounded responses tailored to individual users. In addition, multimodal interaction (particularly through improvements in speech synthesis, prosody, and expressive dialogue) can help bridge the gap between human and AI communication, fostering greater emotional resonance and natural flow. Lastly, immersive environments, including embodied CA and virtual reality settings, may amplify presence, potentially engaging neural and psychological mechanisms typically associated with human-to-human interaction. These innovations must be accompanied by a strong ethical and regulatory foundation. Systems must ensure transparency, informed consent, and compliance with data privacy standards such as GDPR and HIPAA. Crucially, AI should not be viewed as a replacement for psychologists, but as an adaptive and supportive layer within a broader care ecosystem. By aligning technological capabilities with clinical intent, the future of GenAI in mental health may lie in its ability to complement human expertise and meaningfully extend psychological support.

## Introduction

The use of conversational agents (CAs) for adjunctive psychological support seems like an entirely new topic, but some recent research points out that this may already be something well underway (1, 2), to the point that continued hesitation on the part of some clinical institutions about their use may be producing the effect of falling inexorably behind the continuing advances in digital health. As highlighted by Hatch et al. (3), “*mental health experts find themselves in a precarious situation: we must quickly discern the possible destination […] of the AI-therapist train as it may have already left the station*” (p. 13). This suggestion, although it may come across as provocative to someone, shows that Generative Artificial Intelligence (GenAI) for adjunctive psychological support can break through scientific literature, making use of research and data that increasingly demonstrates its effectiveness. Indeed, the debate about the use of CAs to provide user assistance and psychological support is not entirely recent (4, 5). In the mental health context, CAs have already been widely used to act as therapists, counselors, and facilitators (6–8). For example, several years ago, Cristea et al. (9) showed that comparing face-to-face versus computer-based interventions could provide important insights. The authors focused on comparing a chatbot that simulated therapeutic interventions, the “ELIZA” program, with a trained human cognitive behavioral therapist (CBT). The research investigated aspects related to therapeutic performance, such as the effectiveness of the discussion, the correct approach to the problem brought by the patient, and the quality of the therapeutic relationship. The conclusions drawn from the research showed that most evaluators agreed that both therapists were regarded as human people, although with different skills. The only perceived difference between ELIZA and the CBT therapist seemed to lie in the quality of therapeutic performance, but not in any inherent characteristics of the two. Needless to say, things have changed a lot from 2013 to the present, especially in terms of technological advances, so to expect that at present the use of CAs can give better results than those obtained more than a decade ago does not seem to be a far-fetched assumption (10, 11). Rapid advances in Large Language Models (LLMs) such as ChatGPT have generated interest in their potential for clinical applications, sparking both enthusiasm and skepticism. While AI-based interventions aim to improve accessibility and reduce costs, their clinical effectiveness is being tested and the results that have been obtained are opposing (12, 13) but promising (14–16). We could say that today’s question is not whether these tools work, but *how*, *when*, *why,* and *for whom* (17). From a public mental health perspective, this also necessitates consideration of *at what scale* and *within which service delivery models* these technologies can be responsibly integrated. The global treatment gap in low- and middle-income countries remains substantial, with estimates exceeding 80% (18), highlighting the relevance of scalable approaches within stepped-care frameworks, where lower-intensity supports precede or complement face-to-face psychological support. In this broader landscape, AI-enabled tools are sometimes discussed as one possible avenue to support reach and efficiency, though their role depends on context, regulation, and local capacity.

Recent studies have demonstrated that users struggle to distinguish whether responses come from a real clinical professional or a CA. Research shows that regarding common therapeutic factors (therapeutic alliance, empathy, expectations, cultural competence, and therapeutic technique), CAs consistently score higher than mental health professionals themselves (3). However, further findings suggest caution when using CAs. When users are informed about the source of responses, an attribution bias emerges, revealing a technophobic attitude (19). Users tend to attribute positively impressive responses to human therapists while assigning less valuable responses to CAs (3). This phenomenon raises the most challenging question about CA use in psychological support: are we ready to accept help from a tool specifically designed to support us, rather than from someone motivated by professional vocation? In other words: is the recognition we receive from a CA sufficient to make us feel better (20, 21)?

To address these questions, we must move beyond intuitive reactions and subjective biases. A scientific approach requires suspending preconceptions and critically examining evidence (22). Rather than automatically rejecting or embracing AI as an adjunctive psychological support, we need to rigorously evaluate its capabilities, limitations, and potential for meaningful psychological engagement.

Although CAs show great promise in expanding access to psychological support, particularly through scalable and immediate assistance, they also have substantial limitations that require careful consideration. This particularly applies to general-purpose LLMs, such as ChatGPT or Gemini, which are trained on vast, unfiltered internet corpora without clinical supervision or structured psychological knowledge bases. These models, while linguistically fluent, can produce hallucinatory or misleading content and reflect implicit social or cultural biases, posing risks when applied in high-stakes psychological contexts (12). In contrast, mental health-specific GenAI systems trained on curated clinical datasets and optimized for the specific domain offer more structured representations of knowledge, but still face challenges in terms of transparency, interpretability, and generalizability. Furthermore, over-trust on these systems, especially among the most vulnerable or emotionally unstable users, may undermine integrity of the psychological support, highlighting the need for rigorous oversight, explicit scope delimitation, and human supervision in deployment (23). Some fundamental limitations are associated with the quality of the interaction and the ability to effectively facilitate self-reflection, aspects widely recognized as central to psychological support (20). For psychological support, pre-reflective and pre-thematic processes of intercorporeal attunement are essentials (24). This means that when establishing an interaction, the body’s nonverbal communicative work cannot be overlooked. In addition, self-reflection involves the recursive examination of one’s internal landscape, thoughts, emotions, behaviors, and underlying beliefs to develop greater self-awareness and psychological insight. This process is typically catalyzed by human clinicians who can offer empathic attunement, contextual sensitivity, and the capacity to adaptively challenge cognitive distortions. In contrast, AI systems lack non-verbal communication, bodily presence, intentionality, conscious awareness, and metacognitive flexibility, which are critical faculties for guiding clients through the layered complexities of emotional and existential experience. While advanced language models can generate coherent and emotionally resonant responses, they do so without subjective understanding or a genuine grasp of user intent. Furthermore, current CAs are primarily optimized for linguistic coherence and conversational fluency, not for promoting the kind of open-ended inquiry, emotional mirroring, and Socratic questioning that drive reflective growth.

Consequently, while CAs may simulate aspects of the dialogue, they are not yet capable of eliciting insight-oriented transformation. They cannot fully decode subtext, intrapersonal conflict, or nonverbal cues, elements that are essential to identifying maladaptive schemas and fostering internal change. This deficiency limits their effectiveness in encouraging the introspective processes essential for meaningful psychological support.

Despite these limitations, optimizing CAs for psychological support is an ongoing effort.

This perspective article highlights the critical challenges that must be addressed for GenAI to become a viable mental health tool, such as the need for enhancing personalization, multimodal interaction, and immersive environments. We propose a framework to connect these three broad challenges in an epistemic structured model with psychological relevance. The current limitations of CAs can be overcome through targeted optimizations that align AI interventions with established psychological support principles.

## Personalization through psychological domain adaptation and tailored instructions

One of the main limitations of current CAs is their generic nature which lacks personalization and specificity. This limitation impairs their ability to recognize and appropriately respond to complex emotional states and experiences (17). This is also confirmed by a recent perspective (19) stating that generic LLMs in healthcare require domain-specific adaptation. The authors recommend using techniques such as domain-specific tuning, retrieval of verified clinical knowledge, and explicit guardrails to reduce hallucinations, improve factual accuracy, and align results with clinical needs. Considering this, we need to frame personalization on two different, but at the same time complementary, layers: the first shows the personalization as domain-specific knowledge, while the second represents the personalization as tailored response instructions.

*Personalization as domain-specific knowledge*: To improve personalization, we propose to use the back-end Psychological Domain Adaptation (PDA) to supply clinical context and psychological support through three possible mechanisms: grounded retrieval-augmented generation (RAG), fine-tuning, and knowledge bases-backed (for custom GPT). RAG externalizes knowledge at inference time by conditioning responses on vetted clinical/psychological sources and displaying citations. This favors psychological support through psychoeducational uses that require traceability and principled abstention when evidence is lacking. Recent health-information evaluations show that RAG reduces hallucinations and increases appropriate non-response when the reference corpus is reliable (25, 26).

Fine-tuning (including instruction/prompt tuning) internalizes psychological language, support strategies, and response schemas in the model parameters, stabilizing supportive dialogue. Work grounded in emotional-support corpora [e.g., ESConv; (27)] and recent fine-tuning studies for long emotional-support conversations report more consistent strategy use and higher human-rated support quality (28).

Curated knowledge bases (KB-backed custom GPTs) provide the verifiable corpus that assistants index via embeddings/semantic search and are useful when content does not require updates. A recent study demonstrates their potential through SOCRATES (29) for accessible conversational psychological support.

To our knowledge, no study has thus far evaluated the incremental psychological support effect of back-end PDA. However, we believe that this topic is central to refining the use of CAs in adjunctive psychological support contexts. A distinctive feature of GenAI systems is their inherent multilingual capability and the potential for cultural adaptation through cultural-linguistic-specific training (30). This theoretically positions them as tools capable of addressing linguistic and cultural barriers in accessing mental health. However, this would require model training that takes into account culturally specific idioms of distress, help-seeking patterns, and common psychological approaches (25).

*Personalization as tailored response instructions*: This layer specifies how the CA communicates and when it must be handed off. We can define an explicit CA identity (supportive, non-diagnostic, adjunct to care) with a constrained scope. In addition, an empathic response template that prioritizes reflective listening, acknowledgment of uncertainty, and “clarify-before-advice” can be useful with a safety lexicon + intent rules that trigger crisis flows. Concretely, we can maintain a red-flag lexicon (e.g., suicidal or self-harm terms) paired with intent detection. On detection, CA must avoid procedural advice, acknowledge risk, provide region-appropriate crisis options and urgent human escalation, and continue only in a supportive/containment mode. The need for such keyword/intent guardrails is evidenced by evaluations showing that many health CAs still fail to respond appropriately to suicidality prompts [only ~44%, (31)], and by comparative studies of LLMs on suicidality response standards that show good performance at extremes but variability for intermediate-risk queries, underscoring the importance of calibrated prompts and explicit escalation criteria rather than implicit “best effort” behavior (32).

However, operationalizing fine-tuning and RAG in adjunctive psychological support entails concrete technical barriers. First, mental-health–specific corpora remain limited and costly to curate at scale, constraining robust domain adaptation and evaluation. Second, privacy risks are non-trivial: model adaptation and retrieval pipelines can surface or memorize sensitive content, requiring strict de-identification and leakage-mitigation strategies (33). In addition, cultural and linguistic adaptation is an open problem: models trained on majority-language, majority-culture data often fail to generalize to diverse clinical norms and idioms, necessitating localized data and ongoing validation. These constraints imply that personalization must be engineered alongside data governance and culture-aware evaluation, not solely through architectural improvements (34, 35). Lastly, instruction-based tuning that helps models adopt a specific, warmer, and more appropriate tone is essential. Personalization allows systems to recognize uncertainty and seek clarification rather than making assumptions, a critical feature for maintaining ethical GenAI interactions (36).

## Advancing human-AI interaction through spoken and expressive language

Most existing CAs for adjunctive psychological support rely on text-based interactions, limiting their ability to replicate the richness of human conversation. While text-based systems offer accessibility and convenience, spoken dialogue introduces additional dimensions of engagement, particularly through prosody, tone, and rhythm. Research demonstrates that voice-based AI can enhance user trust and emotional connection, making interactions more natural and engaging (37). Furthermore, voice interfaces could theoretically improve accessibility for populations with literacy difficulties or visual impairments (38).

In addition to linguistic fluency, empathetic CAs can leverage affective computation pipelines to infer user state from acoustic-prosodic correlations (e.g., intensity dynamics, spectral slope, jitter/shimmer, pause structure, turn timing) and integrate these signals with lexical/pragmatic features to better tailor responses (39). In psychological support, such recognition of vocal emotions and adaptive prosody have shown promise in increasing perceived empathy and engagement, but they exhibit sensitivity to demographic, cultural, and channel variability, with risks of misclassification in the presence of noise and heterogeneous conversation styles (40). To avoid expectation drift and over-reliance (41), voice systems should combine emotion-based adaptations with calibrated transparency (e.g., safety/uncertainty cues), provide human support, and have cross-language and cross-psychological norm validation.

The effectiveness of synthetic voice depends on its ability to convey warmth, empathy, and dynamically adapt to conversational cues. Current speech synthesis technologies often lack the modulation and affective nuances necessary for effective psychological support communication. Additionally, turn-taking and latency issues can disrupt conversational flow, reducing the AI system’s perceived responsiveness. Addressing these challenges requires integrating advanced speech synthesis models capable of real-time prosodic adjustments, enabling AI to mirror human-like conversational rhythms.

However, the introduction of voice-based AI also raises concerns about user perception. Studies suggest that when AI-generated voices become too like human voices, users may develop unrealistic expectations of the CA’s capabilities, leading to potential disappointment or overdependence (11, 36). Maintaining ethical AI-assisted psychological support requires balancing naturalistic discourse with clear transparency about AI limitations and a simultaneous effort by clinicians and developers.

## The role of immersion in GenAI

In addition to text- and speech-based interactions, the use of CA in Virtual Reality (VR) environments represents a promising frontier in AI psychological support. Immersive technologies have been explored in various mental health applications, particularly exposure therapy and cognitive rehabilitation, demonstrating their potential to improve engagement and clinical outcomes (42, 43).

Three levels of immersion are possible. At the most basic level, an interface can be represented by a CA with a static embodiment (CASE), which provides a visual presence without additional interaction. A more advanced approach involves non-immersive embodied CA (NIECA) that synchronizes lip movements and facial expressions, creating a more dynamic conversational interaction in a non-immersive VR environment. Finally, full immersion in a VR environment enables a simulated dialogue session in which users can interact with immersive embodied CA (IECA). Within this feasibility backdrop, embodiment’s contribution may enhance engagement through presence, potentially supporting adherence and depth of participation (44, 45).

The concept of virtual entity (VE) may expand the potential of AI systems as tools of psychological support. When users perceive IECA as VE within shared virtual spaces, interactions may shift from abstract text exchanges into more naturalistic. Literature suggests that IECA may elicit responses that partly overlap human-to-human social and emotional processes: from the level of brain activation and physiology to subjective feelings of trust, empathy, and connection; whether these signals mediate clinically meaningful change remains to be established (46–48). IECA and NIECA also enable non-verbal communication through gestures, posture, and proxemics, crucial elements often missing in traditional digital interventions. Nevertheless, this embodied experience raises ethical considerations about attachment formation and transference with VE, requiring careful implementation guidelines to maintain appropriate boundaries while leveraging the benefits of embodied presence. While some studies suggest that IECA can reduce social anxiety, improve self-reflection (49), and increase engagement and social closeness, others warn of potential negative effects, including cognitive overload and derealization in vulnerable populations (50). Additionally, the “uncanny valley” phenomenon, where highly realistic but slightly flawed IECA create discomfort, must be carefully considered when designing AI-driven psychological support environments. But beyond the design possibilities, supportive psychological implementation also depends on practical constraints. The total cost of ownership includes the availability of many headsets if used on a large scale, software licenses, and training for psychological staff; these factors determine feasibility and productivity in routine care (43). Moreover, tolerance to VR is uneven: symptoms such as digital motion sickness and discomfort vary depending on users and settings; some users (e.g., those with vestibular problems, migraines, or epilepsy) are not eligible, and home use adds stable connectivity and remote supervision requirements to ensure safety and adherence (51). Current evidence in mental health is still largely confined to controlled trials, so controlled studies, standardized outcomes, and safety monitoring are needed before proceeding to large-scale rollout. Immersion may amplify derealization/dissociation or excessive identification with IECA; therefore, prudent use requires pre-session screening, time limits, gradual exposure, and post-session debriefing to maintain psychological safety (51).

## Theoretical framework: PMVE for psychological support applications

**Figure 1:**
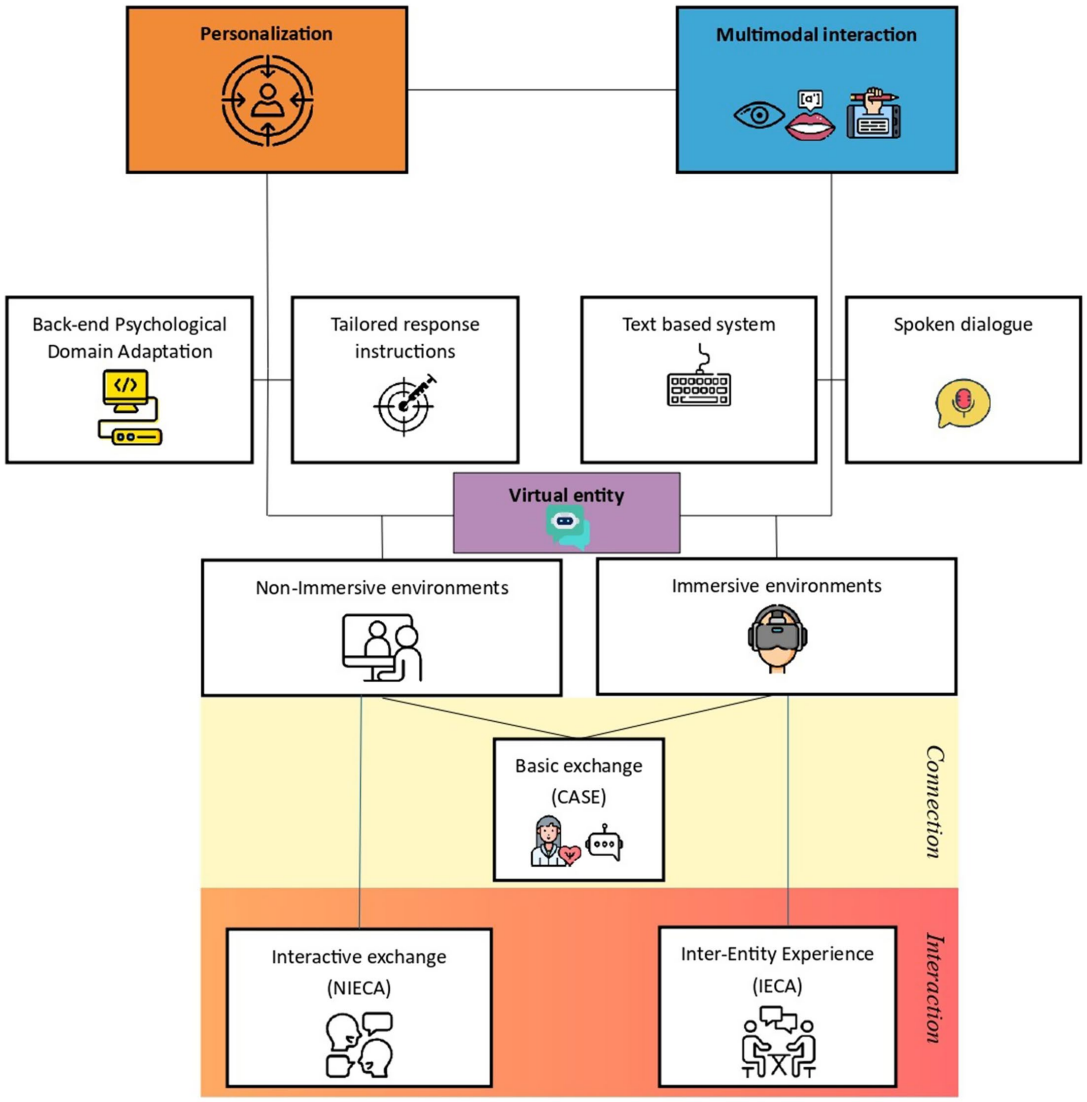
The PMVE framework (Personalization, Multimodality, and Virtual-Entity) for generative AI in psychological support. The model illustrates three interdependent layers (personalization, multimodal communication, and virtual entity) that collectively enhance epistemic alignment, psychological responsiveness, and experiential engagement between users and AI systems. Each layer contributes to transforming virtual entities from purely conversational agents into context-sensitive and psychological support interventions. Icons used in Figure 1 were obtained from Flaticon (www.flaticon.com). Credits by panel are as follows: Multimodal interaction: human eye and headset by Freepik (https://www.flaticon.com/authors/freepik) and mouth illustration by Fitri Handayani (https://www.vecteezy.com/members/amalialuri19948857). Personalization: user profile by Elzicon (https://www.flaticon.com/authors/elzicon). Back-end Psychological Domain Adaptation: Computer by Freepik (https://www.flaticon.com/authors/freepik). Tailored response instructions: precision medicine icon by gravisio (https://www.flaticon.com/authors/gravisio). Text-based system: keyboard icon by Freepik (https://www.flaticon.com/authors/freepik). Spoken dialogue: microphone icon by Fathema Khanom (https://www.freepik.com/author/fathema-khanom). Virtual entity: chatbot icon by Freepik (https://www.flaticon.com/authors/freepik). Non-Immersive environments: Online meeting by user21718592 (https://www.freepik.com/author/user21718592/icons). Immersive environments: VR Headset by Freepik (https://www.flaticon.com/authors/freepik). Basic exchange (CASE): psychologist and bot icons by Freepik (https://www.flaticon.com/authors/freepik). Interactive exchange (NIECA): chat icon by narak0rn (https://www.flaticon.com/authors/narak0rn). Inter-Entity Experience (IECA): conversation icon by Prosymbols Premium (https://www.flaticon.com/authors/prosymbols-premium).

In light of the above considerations and evidence, we propose a theoretical framework (see Figure 1) aimed at integrating the three challenges (personalization, multimodal interaction, and immersive environments) as a prospective solution to the limitations of general-purpose CAs and a pathway toward their responsible and personalized integration in adjunctive psychological support.

We refer to this three-layer framework as the PMVE framework (Personalization, Multimodality and Virtual-Entity) that links (i) epistemic alignment, (ii) multimodal communication, and (iii) graded immersion to human-AI interaction.

We next unpack each layer in turn.

Personalization ensures epistemic consistency by aligning the system’s outputs with domain-specific psychological principles and dynamically adapting responses to the user’s profile. Tailored response instructions and the back-end PDA translate epistemic structures into individualized practices, ensuring that the system’s adaptive responses maintain psychological supportive relevance and contextual sensitivity.

Text-based messages and voice responses (TTS) constitute the main channels of human-VE communication, establishing the linguistic substrate through which psychological supportive processes unfold. Building on this foundation, multimodal interaction expands the communicative spectrum by incorporating sensory and contextual dimensions that enable a more natural, embodied, and immersive engagement.

The subsequent layer, encompassing immersive environments and inter-entity experience, extends interaction into virtual contexts, where users engage with IECA that co-construct and sustain the setting. Notably, this opportunity allows for a level of exchange that is not only interactive, but experiential. We argue that the degree of user immersion and perceived interactivity increases systematically with each level of embodiment. This progression can be further conceptualized in terms of the intensity of the underlying bond: from basic exchange, as the mere establishment of a communicative link (e.g., with a CASE); to interactive exchange, denoting reciprocal and dynamic exchange (e.g., with a NIECA); and finally, to a sustained and co-constructed inter-entity experience (e.g., with a IECA). Each level corresponds to an increasing depth of engagement and mutual influence, reflecting the transition from potential connectivity to fully embodied experience. A disembodied interaction remains purely symbolic, while an immersive inter-entity experience establishes a fully embodied, spatially co-located interaction. This difference highlights the transition from cognitive to sensorimotor forms of engagement in human-VE communication. The degree of embodiment and immersion directly influences the potential of human-VE interactions. Lower levels (e.g., disembodied or static representations) may support basic cognitive interventions but provide limited emotional resonance. Conversely, higher levels, particularly immersive, enable multisensory engagement and co-presence, fostering empathy, behavioral modeling, and embodied learning.

**Figure 2:**
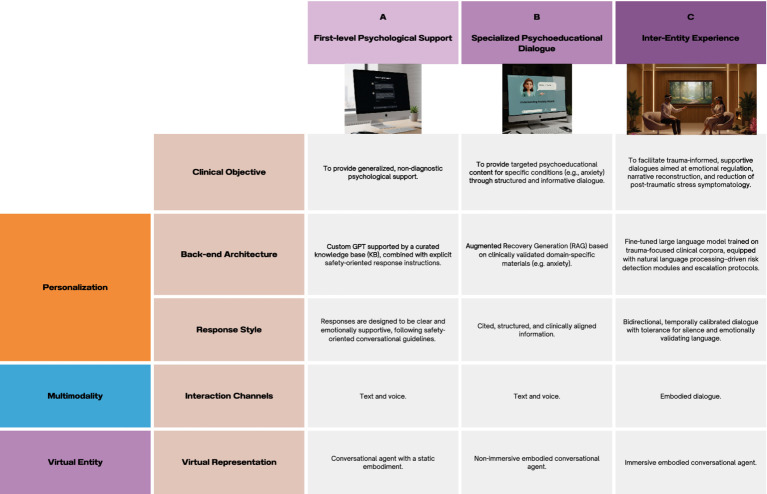
Three examples of possible application scenarios from the PMVE framework. **(A)** Generalized psychological support by a conversational agent (CA) with a static embodiment; **(B)** psychoeducation on anxiety via RAG and non-immersive embodied CA; **(C)** inter-entity experience for post-traumatic stress disorder with immersive embodied CA. This figure conceptualizes how increasing levels of immersion are correlated with greater presence and emotional resonance. The transition from connection to interaction, and ultimately, experience, reflects the progression from symbolic to dynamic modes of human-AI interaction. Applications vary based on purpose.

The PMVE framework suggests that the experiential depth of the interface can modulate outcomes in digital settings for psychological support (see Figure 2). This is why it becomes crucial to determine which type of setting may be most beneficial for a VE-based intervention. At the same time, it is important to acknowledge that such settings should be precisely tailored to the user’s characteristics and goals. Consequently, immersive environments, despite their greater potential for fostering meaningful experiences, will not always represent the most appropriate or effective solution. Collectively, these components articulate an epistemically structured model that bridges psychological, theoretical, and technological domains, fostering an integrative approach to psychological support in digital and immersive settings.

## Toward ethical and effectiveness GenAI for psychological support

The integration of GenAI into psychological support marks a pivotal evolution in the field, offering both unprecedented opportunities and significant ethical and clinical challenges. On one hand, GenAI holds immense promise: it can enhance accessibility, reduce systemic delays, and lower the cost of care, particularly for underserved and marginalized populations. On the other hand, current implementations, especially those relying solely on text-based interactions, often fail to guarantee the fundamental conditions for meaningful psychological support. This, indeed, includes the capacity to facilitate self-reflection, provide emotionally attuned and context-sensitive responses, and respect the user’s cognitive and emotional bandwidth. Without these elements, AI-driven interventions risk being experienced as mechanistic, impersonal, or even detrimental.

Emerging research in IECA suggests that more relationally aware systems may begin to bridge this gap (52). Functional neuroimaging studies have demonstrated that emotionally responsive NIECA and IECA can engage neural circuits implicated in social bonding, such as the medial prefrontal cortex, insula, and temporoparietal junction, areas fundamental to empathy, mentalizing, and perspective-taking (46–48, 53). Moreover, NIECA and IECA that mirror human nonverbal cues or engage in empathic dialogue have been shown to elicit affective bonds, encourage emotional disclosure, and even predict outcomes in digital mental health settings. These findings lend empirical support to the notion that AI, when thoughtfully designed, can transcend transactional dialogue and foster a sense of presence. However, even as AI systems become more sophisticated, their psychological validity remains contingent on continuous oversight, clear role definition, and transparent communication regarding the system’s capabilities and limitations (23).

In realizing this potential, adherence to clinical integrity, ethical transparency, and user safety must remain non-negotiable. To render these measures genuinely actionable in a psychological-support CA, the governance architecture should weave data protection, transparency, jurisdiction-specific incident readiness, lifecycle accountability, and meaningful clinical oversight into a coherent operational design. At the data-governance layer, any secondary use of conversational traces is preceded by a documented assessment of purpose compatibility and a clear articulation of the lawful basis for processing special-category data; safeguards associated with Article 89 of the GDPR (data minimization and pseudonymization) are applied ex ante and the resulting determinations are reflected in user notices in line with EU guidance on further processing (54). The user interface operationalizes transparency obligations by making explicit that the interlocutor is an AI system, by providing an intelligible indicator whenever emotion-recognition functionality is active, and by unambiguously labeling synthetic outputs; each of these transparency events is durably logged to support audit and regulatory review, consistent with Article 50 of the EU AI Act (55). Deployments aimed at the United States are scoped at the outset to determine whether the arrangement falls within HIPAA, including whether a covered-entity or business-associate relationship necessitates a Business Associate Agreement; where HIPAA does not apply, breach-response workflows are aligned with the Federal Trade Commission’s Health Breach Notification Rule, as amended and effective 29 July 2024, and harmonized with Office for Civil Rights guidance on individual rights (62). Lifecycle accountability treats the CAs as software within the ambit of the new EU Product Liability Directive, maintaining versioned model cards, safety-relevant change logs, and secure update/rollback policies capable of addressing post-sale defects, including those introduced by model updates or continuous learning, thereby preserving traceability and remedial agility (56). Human-in-the-loop oversight qualified clinicians must be able to inspect inputs and plain-language rationales, monitor for anomalies, override or stop the system, and remain accountable for use; meeting the EU AI Act’s human-oversight obligations and the FDA’s “Non-Device CDS” criterion that clinicians independently review the basis for recommendations. These measures turn abstract compliance into concrete UX patterns, governance artifacts, and operational controls that protect users while preserving the value of psychological support.

## From framework to evaluation: evidence gaps and conclusions

While the PMVE framework draws on convergent findings from affective computing, human-computer interaction, and clinical psychology, the evidence base for embodied and immersive implementations remains limited. As far as we know, no randomized controlled trials have directly compared personalization strategies (RAG versus fine-tuning) using patient-centered outcomes in psychological contexts (25). Voice-based affective computing studies remain predominantly correlational; the causal impact of adaptive prosody on psychological change has not been established in longitudinal designs (57).

Neural activation patterns elicited by IECA demonstrate engagement of social cognition networks (46), yet these findings do not establish psychological efficacy. As far as we know, no studies have demonstrated that IECA produce superior outcomes to text-based modalities in adequately controlled trials with standardized mental health endpoints. The potential for adverse effects, including attachment formation, parasocial over-reliance, or exacerbation of dissociative symptoms, has been noted qualitatively (58) but lacks systematic prospective monitoring. This matters at system level because adverse events and over-reliance are not only individual risks but also implementation risks that can affect service demand, escalation burden, and trust in digital pathways if not monitored prospectively. WHO guidance on digital interventions (59) emphasizes that digital tools should be implemented as part of functioning health systems, with attention to benefits, harms, feasibility, and equity rather than as standalone substitutes. From a preventive perspective of possible adverse events, it must be considered that AI-based psychological support systems are not appropriate for all clinical presentations and may pose specific risks to vulnerable populations. For example, trauma-related dissociative disorders present heightened risk in immersive environments, where spatial presence and perceptual realism may trigger dissociative episodes or emotional flooding (51). Furthermore, individuals with psychotic features, severe mania, or eating disorders require specialized safeguards, as general-purpose systems may inadvertently reinforce maladaptive cognitions or exacerbate perceptual disturbances (60, 61).

The PMVE framework should be considered as a structured roadmap ready for evaluation. Accordingly, we propose PMVE as an incremental, evidence-proportionate pathway (see Figure 2), where the three levels differ not only in modality but in their expected health-system contribution: lower-intensity modes can broaden reach and reduce wait-time pressure, hybrid modes support continuity and triage, and inter-entity experiences are reserved for protocolized, supervised use where added value justifies higher cost and risk. However, practical barriers to population-wide implementation include infrastructure requirements (reliable bandwidth, device access) that remain unequally distributed, particularly in resource-constrained settings.

The next step is therefore straightforward: move from plausible mechanisms to measurable clinical and implementation endpoints, so that PMVE’s benefits, and its boundaries, can be established with the same rigor expected of any intervention intended for real-world mental health systems. In this light, GenAI should be understood not as a standalone solution, but as a complementary ally, a tool best deployed when human presence is unavailable, and always in service of enhancing, not replacing, the human connection at the heart of mental health care. This perspective article outlines a plausible path (personalization, multimodality, and inter-entity experience) through which GenAI systems could represent adjunctive psychological support; these possibilities deserve testing. Future research should rigorously evaluate their effectiveness in mental health care.

## Data Availability

The original contributions presented in the study are included in the article/supplementary material, further inquiries can be directed to the corresponding author.

## Glossary

Assistants API: An API-defined agent configuration object that binds (i) a base model, (ii) developer-provided instructions, and (iii) an enabled set of tools and tool resources (e.g., file_search vector stores, code interpreter), and that executes over a persistent Thread by creating a Run, during which the assistant may invoke tools and integrate their outputs into the response.
Conversational agent (CA): An interactive artificial intelligence system designed to communicate with a user in natural language (text and/or speech) to provide information, assistance, or social interaction. If it’s text-based only, it’s called a chatbot.
Conversational agent with static embodiment (CASE): Conversational agent that has a static visual representation (avatar or profile picture) that is not animated and non-interactive in real time.
Curated knowledge base (KB): an externally maintained collection of domain content that is selected, cleaned, and versioned to serve as a non-parametric store of knowledge that can be accessed explicitly by a model (e.g., via a dense vector index) rather than being encoded only in model parameters.
Embodied Conversational Agent (ECA): A CA represented by an on-screen character (or avatar) that uses verbal and nonverbal behaviors (e.g., gaze, gesture) to support face-to-face–like interaction. It can be deployed as a Non-Immersive ECA (NIECA; presented on a conventional 2D display) or as an Immersive ECA (IECA; situated in an immersive virtual environment).
Fine-tuning: Adapting a pretrained model to a downstream task/domain by continuing training on task-specific data while updating (most or all) model parameters, yielding a task-specialized model instance.
Grounded retrival-augmented generation (RAG): a retrieval-augmented generation setup in which a model retrieves relevant passages from an external corpus at inference time and uses the retrieved evidence to support and constrain its output, aiming to improve provenance/verifiability and reduce unsupported generation (hallucinations).
Virtual embodiment: The process/illusion by which a user maps their body schema onto a virtual body (avatar) and comes to experience that virtual body as an extension of the self, typically supported by afferent (e.g., visuo–tactile) and/or sensorimotor correspondences between the user’s physical body and the avatar’s body.
Virtual entity: Terminology proposed by the authors to describe how the combination of personalization and multimodality creates something different from a generic conversational agent, whose characteristics recall those of a non-human virtual entity.
Virtual environment: A computer-generated environment that includes a virtual space plus its content and interaction rules, enabling users to perceive and act within it as an interactive world.
Virtual space: A computer-generated spatial framework that defines where variables can be located (positions, distances, directions) within a digital system.
